# Near Infrared-Emitting Cr^3+^/Eu^3+^ Co-doped Zinc Gallogermanate Persistence Luminescent Nanoparticles for Cell Imaging

**DOI:** 10.1186/s11671-018-2477-6

**Published:** 2018-02-27

**Authors:** Qiaoqiao Wang, Shuyun Zhang, Zhiwei Li, Qi Zhu

**Affiliations:** 1 Key Laboratory for Anisotropy and Texture of Materials (Ministry of Education), Northeastern University, Shenyang, Liaoning 110819 China; 2 Institute of Ceramics and Powder Metallurgy, School of Materials Science and Engineering, Northeastern University, Shenyang, Liaoning 110819 China

**Keywords:** Near infrared (NIR)-emitting, Persistent luminescent, Bioimaging, Spinel structure, Nanoparticles

## Abstract

Near infrared (NIR)-emitting persistent luminescent nanoparticles have been developed as potential agents for bioimaging. However, synthesizing uniform nanoparticles with long afterglow for long-term imaging is lacking. Here, we demonstrated the synthesis of spinel structured Zn_3_Ga_2_Ge_2_O_10_:Cr^3+^ (ZGGO:Cr^3+^) and Zn_3_Ga_2_Ge_2_O_10_:Cr^3+^,Eu^3+^ (ZGGO:Cr^3+^,Eu^3+^) nanoparticles by a sol-gel method in combination with a subsequent reducing atmosphere-free calcination. The samples were investigated via detailed characterizations by combined techniques of XRD, TEM, STEM, selected area electron diffraction, photoluminescence excitation (PLE)/photoluminescence (PL) spectroscopy, and temperature-dependent PL analysis. The single-crystalline nanoparticles are homogeneous solid solution, possessing uniform cubic shape and lateral size of ~ 80–100 nm. Upon UV excitation at 273 nm, ZGGO:Cr^3+^,Eu^3+^ exhibited a NIR emission band at 697 nm (^2^E → ^4^A_2_ transition of distorted Cr^3+^ ions in gallogermanate), in the absence of Eu^3+^ emission. NIR persistent luminescence of the sample can last longer than 7200 s and still hold intense intensity. Eu^3+^ incorporation increased the persistent luminescence intensity and the afterglow time of ZGGO:Cr^3+^, but it did not significantly affect the thermal stability. The obtained ZGGO:Cr^3+^,Eu^3+^-NH_2_ nanoparticles possessed an excellent imaging capacity for cells in vitro.

## Background

Persistent luminescent materials can emit for a long time up to hours after the stoppage of excitation [1]. Mainly due to their great research interests, the phosphors have been commercialized as night or dark environment vision materials for a wide range of applications such as security signs, emergency route signage, identification markers, or medical diagnostics [2]. The typical long-persistent phosphors are the commercialized primary color emitters, such as the red Y_2_O_2_S:Eu^3+^,Mg^2+^,Ti^4+^ or CaS:Eu^2+^,Tm^3+^,Ce^3+^ [3, 4], the green SrAl_2_O_4_:Eu^2+^,Dy^3+^ or MgAl_2_O_4_:Mn^2+^ [5, 6], and the blue CaAl_2_O_4_:Eu^2+^,Nd^3+^ or SrMgSi_2_O_6_:Eu^2+^,Dy^3+^ [7, 8] phosphors. Although many successes have been made in visible persistent phosphors, the investigation and development of persistent phosphors in the near infrared (NIR) region (~ 700–2500 nm) are insufficient. In recent years, the potential applications of persistent phosphors showing red or NIR luminescence have expanded from night-vision security signs to in vivo imaging systems [1, 9, 10].

Persistent luminescent materials with attached photosensitizers as an in vivo agent were firstly tried by Chen and Zhang for photodynamic therapy [11]. Then, Scherman et al. reported a milestone work on in vivo bioimaging with the NIR-emitting phosphor of Ca_0.2_Zn_0.9_Mg_0.9_Si_2_O_6_:Eu^2+^,Mn^2+^,Dy^3+^ [12]. Soon afterwards, two new NIR-emitting phosphors of CaMgSi_2_O_6_:Eu^2+^,Mn^2+^,Pr^3+^ and Ca_2_Si_5_N_8_:Eu^2+^,Tm^3+^ with improved performance have been developed by the same group [13, 14]. Recently, Cr^3+^-doped gallate persistent phosphors with NIR emission and long afterglow, including spinel ZnGa_2_O_4_:Cr^3+^ and their variants, such as Zn_3_Ga_2_Ge_2_O_10_:Cr^3+^, Zn_3_Ga_2_GeO_8_:Cr^3+^,Yb^3+^,Er^3+^, and ZnGa_2 − *x*_(Ge/Sn)_*x*_O_4_:Cr^3+^, were prepared by a solid-state method [1, 9, 10, 15–21]. The ceramic-disc samples exhibited afterglow time up to 360 h at the NIR region, but the bulky materials are unsuitable for in vivo bioimaging. NIR-emitting long-persistent luminescent nanoparticles of ZnGa_2_O_4_:Cr^3+^ [22, 23], ZnGa_2_O_4_:Cr^3+^,Sn^4+^ [19–21], and Zn_2.94_Ga_1.96_Ge_2_O_10_:Cr^3+^,Pr^3+^ [9] were synthesized by a sol-gel method in combination with a subsequent reducing atmosphere-free calcination. The persistent luminescence of the nanoparticles powder exhibits bright NIR luminescence in the biological transparency window with a superlong afterglow time. PEGylation greatly improves the biocompatibility and water solubility of the nanoparticles, which hold great potential for long-term in vivo bioimaging application with high SNR without the need for in situ excitation. It is believed that ions selected from a group consisting of alkaline earth ions, lanthanide ions, and Li^+^ co-doping with Cr^3+^ in zinc gallate and zinc gallogermanate would yield remarkable NIR persistent luminescence [1]. Eu^3+^ in oxide hosts always exhibits a red emission at ~ 700 nm arising from the ^5^D_0_-^7^F_4_ intra-4*f* electronic transition upon short UV excitation into the charge transfer (CT) band at 250 nm [24]. On the other hand, Cr^3+^ is a favorable luminescent center in solids because of its narrowband emissions (usually at 700 nm) due to the spin-forbidden ^2^E-^4^A_2_ transition, or a broadband emission (650–1600 nm) due to the spin-allowed ^4^T_2_-^4^A_2_ transition [1, 20]. In view of these, Cr^3+^/Eu^3+^ co-doped zinc gallate and zinc gallogermanate would yield intense NIR persistent luminescence, owing to that the charge transfer band (CTB) of O^2−^-Eu^3+^ overlaps with the CTB of O^2−^-Ga^3+^, and the emission at ~ 700 nm from ^5^D_0_-^7^F_4_ transition of Eu^3+^ overlaps with that from ^2^E-^4^A_2_ transition of Cr^3+^. Furthermore, Eu^3+^ ions substituting for Ga^3+^ ions in distorted octahedral sites may yield suitable host crystal-field strength around Cr^3+^ ions, thus affecting the NIR emission. In this work, Zn_3_Ga_2_Ge_2_O_10_:Cr^3+^,Eu^3+^ (termed as ZGGO:Cr^3+^,Eu^3+^) nanoparticles were synthesized by a sol-gel method in combination with a subsequent reducing atmosphere-free calcination, which would be used as the promising nanoprobes for future bioimaging. The samples were investigated via detailed characterizations by combined techniques of X-ray diffractometry (XRD), transmission electron microscopy (TEM), STEM, selected area electron diffraction (SAED), photoluminescence excitation (PLE)/photoluminescence (PL) spectroscopy, and temperature-dependent PL analysis. In the following sections, we report the synthesis, characterization, and application of the ZGGO:Cr^3+^,Eu^3+^ nanoparticles.

## Experimental

### Synthesis

The starting metal sources are Zn(NO_3_)_2_·6H_2_O, Cr(NO_3_)_3_·9H_2_O, Ga_2_O_3_, Eu_2_O_3_, and GeO_2_ were all 99.99% pure products purchased from Sinopharm (Shanghai, China). The other reagents are of analytical grade and were purchased from Shenyang Chemical Reagent Factory (Shenyang, China). Zn(NO_3_)_2_·6H_2_O and Cr(NO_3_)_3_·9H_2_O were dissolved in deionized water. Ga_2_O_3_ and Eu_2_O_3_ were dissolved in nitric acid solution. GeO_2_ and ethylenediaminetetraacetic acid (EDTA) were dissolved in dilute ammonium hydroxide. To the mixture solution, EDTA solution was slowly added without any precipitation, and the molar ratio of total metal ions to EDTA was maintained at 1:2. The atom molar ratio of Zn:Ga:Ge:Cr:Eu was fixed to be 3:1.984:2:0.01:0.006. The final solution was vigorously stirred at room temperature for 1 h, then heated in an oven at 85 °C for the slow evaporation of water until the solution became a sol that finally became a gel. The obtained gel was heated at 200 °C for 3 h to form black porous materials. Finally, the porous materials were ground and annealed under flowing O_2_ gas (200 mL/min) at selected temperatures for 2 h.

### Surface Functionalization

The ZGGO:Cr^3+^,Eu^3+^ powder was ground for 30 min, then 150 mg of the obtained sample was added into 50 mL of 0.1 mol/L NaOH solution. After sonication for 1 h, the suspension was vigorously stirred for 24 h at room temperature. The resulting colloid solution was centrifuged at 1000 rpm for 10 min to remove large size particles, and the supernatant was centrifuged at 10,000 rpm for 10 min to collect the precipitate. The as-obtained ZGGO:Cr^3+^,Eu^3+^-OH nanoparticles were washed with deionized water three times.

Ten milligrams of ZGGO:Cr^3+^,Eu^3+^-OH nanoparticles was dispersed in 4 mL of dimethylformamide (DMF) with the assistance of sonication for 10 min. Then, 40 μl of 3-aminopropyl-triethoxysilane (APTES) was added under vigorous stirring for 24 h at room temperature. The as-obtained ZGGO:Cr^3+^,Eu^3+^-NH_2_ nanoparticles were collected by centrifugation at 10,000 rpm for 10 min and washed with DMF three times to remove unreacted APTES.

### Cell Imaging

Hek293T cells were cultured in DMEM with 10% FBS and seeded in 35-mm culture dishes for 2 h in a CO_2_ incubator. The as-obtained ZGGO:Cr^3+^,Eu^3+^-NH_2_ nanoparticles were dispersed in cell medium (50 mg/mL), which were excited for 10 min by a 254-nm UV lamp and then moved to culture dishes treated for 1 h. After removing the cell medium, 0.1 mL of 1% formaldehyde-PBS was added and the cells were stained with 0.5 mL DAPI dye in the dark for 10 min. Finally, the cells were washed with PBS several times for further characterization.

All studies involving animals were approved by the university animal care and use committee.

### Characterization Techniques

Phase identification was performed by XRD (model SmartLab; Rigaku, Tokyo, Japan) operating at 40 kV/40 mA using nickel-filtered Cu Kα radiation and a scanning speed of 6.0° 2*θ*/min. Morphologies of the products were observed via TEM (model JEM-2000FX; JEOL, Tokyo). Photoluminescence of the phosphors was analyzed with an FP-8600 fluorospectrophotometer (JASCO, Tokyo). The persistent luminescence signals were obtained using Horiba JY FL3-21. The afterglow decay images were recorded in a dark room using a Kodak In-Vivo Imaging System FX Pro. The cell imaging was conducted by a laser scanning confocal microscope (LEICA TCS SP2, Germany).

All studies involving animals were approved by the university animal care and use committee.

## Results and Discussion

**Fig. 1:**
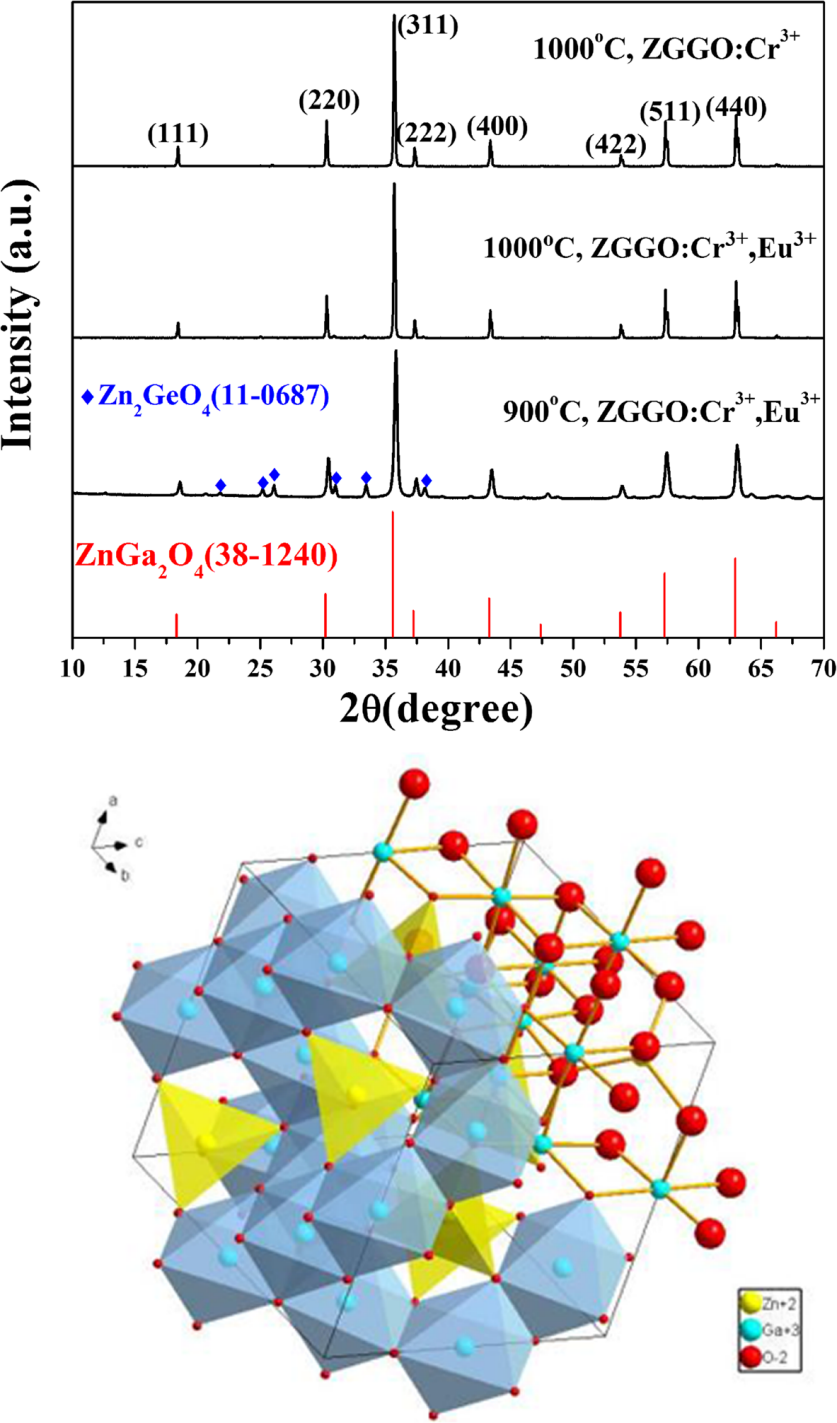
XRD patterns of the as-prepared ZGGO:Cr^3+^ and ZGGO:Cr^3+^,Eu^3+^ and the crystal structure of spinel ZnGa_2_O_4_

The phase purity of the samples was first investigated by XRD. Figure 1 (up) shows the XRD patterns of the as-prepared ZGGO:Cr^3+^ and ZGGO:Cr^3+^,Eu^3+^ calcined at 1000 °C, which were identified along with the spinel structured Zn_3_Ga_2_Ge_2_O_10_ [1, 9]. The crystal structure of Zn_3_Ga_2_Ge_2_O_10_ is the same with that of ZnGa_2_O_4_ (JCPDS No. 38-1240), which is the solid solution of ZnGa_2_O_4_ and Zn_2_GeO_4_. In the structure of Zn_3_Ga_2_Ge_2_O_10_, Ge plays the role of substitution of Ga, conducive to the formation of traps, while ZnGa_2_O_4_ is the dominant crystal structure [1]. There are two kinds of cations in one-unit cell; Zn^2+^ and Ga^3+^ are surrounded by four and six oxygen anions forming a tetrahedron and an octahedron, respectively (Fig. 1, below). Calculations from the diffraction data yielding the cell constants for ZGGO:Cr^3+^ are *a* = *b* = ~ 0.8335 nm, close to that of spinel ZnGa_2_O_4_ (*a* = *b* = ~ 0.8335 nm, JCPDS No. 38-1240). Due to the larger ionic radii of Eu^3+^ (for sixfold coordination, ${r}_{{\mathrm{Eu}}^{3+}}$ = 0.0947 nm and ${r}_{{\mathrm{Ga}}^{3+}}$ = 0.062 nm) [25], a larger value of *a* = *b* = ~ 0.8336 nm was observed for ZGGO:Cr^3+^,Eu^3+^. Profile broadening analysis of the (311) Bragg reflection was conducted by applying the Scherrer equation for average crystallite sizes of 83 ± 6 nm for ZGGO:Cr^3+^ and ZGGO:Cr^3+^,Eu^3+^ samples. In Fig. 1 (up), we also find that the resulting products calcined at 900 °C are the mixture of spinel phase (JCPDS No. 38-1240) and rhombohedral one (JCPDS No. 11-0687), indicating a calcination temperature of ≥ 1000 °C is needed for yielding spinel Zn_3_Ga_2_Ge_2_O_10_ in a phase pure form.

**Fig. 2:**
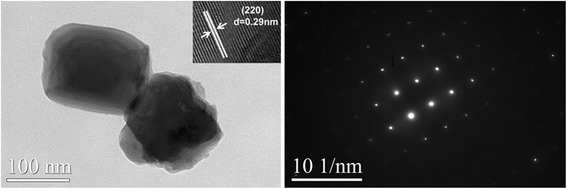
TEM, HR-TEM (left) image, and SAED pattern (right) of the ZGGO:Cr^3+^,Eu^3+^ nanoparticles

**Fig. 3:**
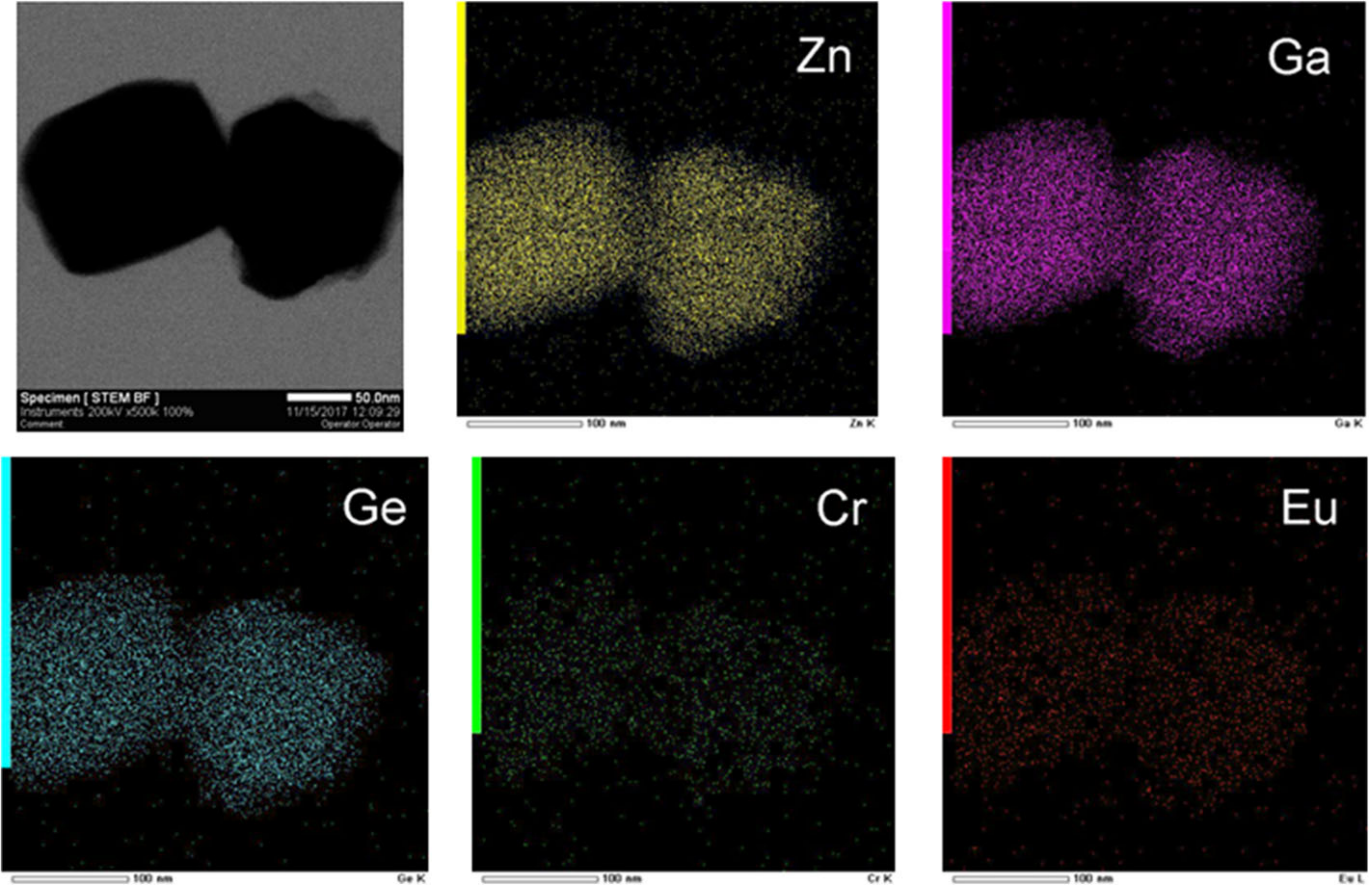
STEM particle morphology (bright field image, the first picture) and elemental mapping of ZGGO:Cr^3+^,Eu^3+^ nanoparticles

Figure 2 (left) shows the TEM morphology for ZGGO:Cr^3+^,Eu^3+^ particles, clearly indicating that they entirely consist of cubic particles, with lateral sizes of ~ 80–100 nm. The sharp corners and the well-resolved lattice fringes suggest their excellent crystallinity, while the spacings of ~ 0.29 nm correspond well to the (220) plain of spinel structured ZnGa_2_O_4_ (*d*(220) = ~ 0.29 nm, JCPDS No. 38-1240) (inset in Fig. 2). Because the particle sizes are close to the average crystallite sizes calculated from the XRD data, the obtained samples may be single crystalline. The SAED analysis (Fig. 2 (right)) further confirmed that the nanoparticles under analysis are of single crystalline. The nanoparticles investigated here are direct solid-state solutions rather than a mechanical mixture. Elemental mapping of Zn, Ga, Ge, Cr, and Eu provides evidence of this solid solution, as revealed in Fig. 3 for ZGGO:Cr^3+^,Eu^3+^. Not only does each particle contain Zn, Ga, Ge, Cr, and Eu, but all elements are evenly distributed among the particles.

**Fig. 4:**
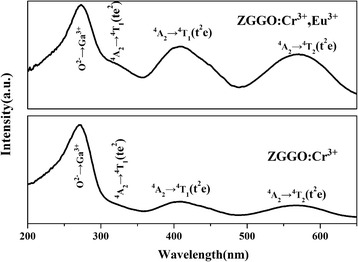
Photoluminescence excitation (PLE) spectra of ZGGO:Cr^3+^ and ZGGO:Cr^3+^,Eu^3+^ powders

**Fig. 5:**
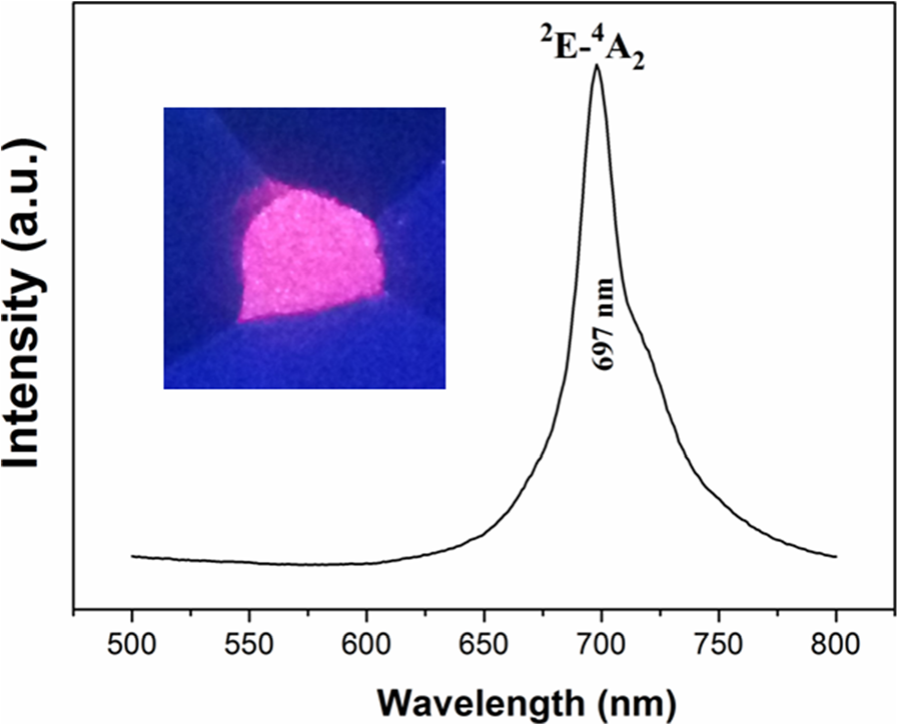
Photoluminescence (PL) spectra of ZGGO:Cr^3+^,Eu^3+^ powders

Figure 4 shows the excitation spectra of the ZGGO:Cr^3+^ and ZGGO:Cr^3+^,Eu^3+^ powder at room temperature. The excitation spectrum monitored at 697 nm covers a very broad spectral region (from 200 to 650 nm) and consists of four main excitation bands peaking at 273, 328, 410, and 569 nm, respectively. The excitation band at 273 nm is ascribed to the charge transfer band of O^2−^-Ga^3+^ in ZnGa_2_O_4_ host, while the later bands originate from the inner transitions of Cr^3+^, including the 328-nm band originating from the ^4^A_2_ → ^4^T_1_(*te*^2^) transition, the 410-nm band originating from the ^4^A_2_ → ^4^T_1_(*t*^2^*e*), and the 569-nm band originating from the ^4^A_2_ → ^4^T_2_(*t*^2^*e*) [19, 20]. Incorporation of Eu^3+^ did not appreciably alter the positions of the PLE bands but significantly increased the intensities of the inner transitions of Cr^3+^, with *I*_410_/*I*_273_ increasing from 0.18 to 0.56. The above results indicate that Eu^3+^ incorporation is conducive to the visible light excitation. However, the strongest excitation band at 273 nm also revealed that the charge transfer band of O^2−^-Ga^3+^ is the most effective excitation wavelength. Excitation of the powder at 273 nm gave a NIR emission band at 697 nm (Fig. 5) due to the ^2^E → ^4^A_2_ transition in distorted Cr^3+^ ions in gallogermanate, in the absence of Eu^3+^ emission.

**Fig. 6:**
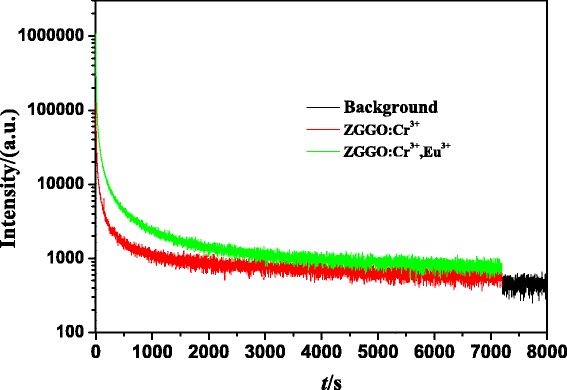
NIR persistent luminescence decay curves of ZGGO:Cr^3+^ and ZGGO:Cr^3+^,Eu^3+^ powders monitored at 697 nm after 254 nm UV light illumination for 5 min

**Fig. 7:**
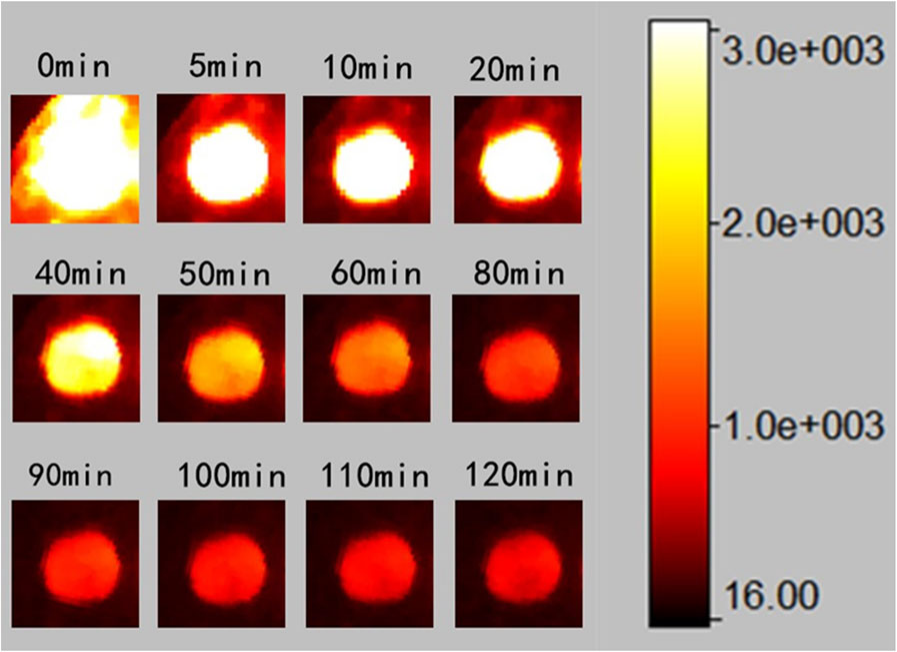
NIR afterglow decay images of ZGGO:Cr^3+^,Eu^3+^ powders obtained by a Kodak In-Vivo Imaging System FX Pro at different times after stopping UV irradiation

The NIR persistent luminescence decay curves of ZGGO:Cr^3+^ and ZGGO:Cr^3+^,Eu^3+^ nanoparticles were monitored at 697 nm after 254 nm UV light illumination (xenon lamp as the light source) for 5 min at room temperature as shown in Fig. 6. The result demonstrates that the NIR persistent luminescence of the ZGGO:Cr^3+^ sample can last longer than 7200 s and still hold appreciable intensity. The persistent luminescence intensity of ZGGO:Cr^3+^,Eu^3+^ increases with the incorporation of Eu^3+^ ion. It is believed that lanthanide ions co-doping with Cr^3+^ in zinc gallogermanate would yield remarkable NIR persistent luminescence, because of its important role in increasing the amount of anti-site defects which is the responsible for persistent luminescence of Cr^3+^ in zinc gallogermanate host [1]. On the other hand, NIR persistent luminescence of ZGGO:Cr^3+^,Eu^3+^ sample can last longer than that of ZGGO:Cr^3+^, indicating that Eu^3+^ incorporation can increase the afterglow time. Figure 7 shows NIR afterglow decay images of ZGGO:Cr^3+^,Eu^3+^ powders obtained by a Kodak In-Vivo Imaging System FX Pro at different times after stopping UV irradiation, further confirming that the afterglow can last longer than 120 min and keep an intense NIR emission intensity.

**Fig. 8:**
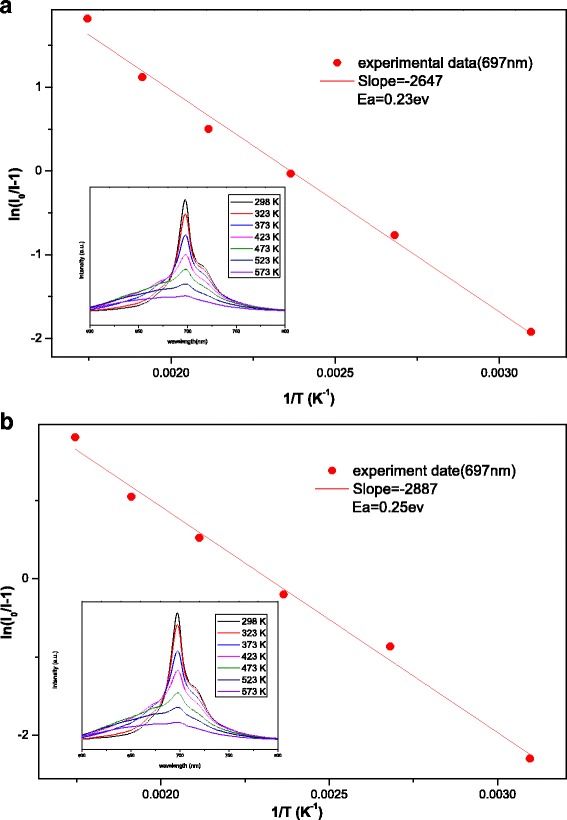
Activation energy of thermal quenching for emission bands in **a** ZGGO:Cr^3+^ and **b** ZGGO:Cr^3+^,Eu^3+^ powders. The insets show the corresponding temperature dependence of PL spectra from 298 to 573 K

For evaluating the performance of phosphor application, especially for high-power applications, thermal stability is a key parameter. To evaluate the thermal quenching behavior of the phosphors in this work, the PL spectra were analyzed at temperatures ranging from 298 to 573 K (Fig. 8). For all samples, elevating the temperature yielded a decrease in the emission intensities at 697 nm. To obtain a more comprehensive picture of the thermal quenching behavior and estimate the value of its activation energy (*E*_a_), the Arrhenius equation (Eq. (1)) was employed as follows [26–28]:

1 $$ {I}_{\mathrm{T}}=\frac{I_0}{1+c\exp \left(-\frac{E_{\mathrm{a}}}{kT}\right)} $$

where *I*_0_ and *I*_T_ are the intensities of the initial and final temperatures, respectively; *c* is the rate constant; *E*_a_ is the activation energy; and *k* is the Boltzmann constant (8.629 × 10^−5^ eV K^−1^). Figure 8 shows the In(*I*_0_ / *I*_T_ − 1) vs 10,000 / *T* relationship lines for the emission band centered at 697 nm for ZGGO:Cr^3+^ and ZGGO:Cr^3+^,Eu^3+^. Similar activation energies were calculated: *E*_a_ = 0.23 eV for ZGGO:Cr^3+^ and *E*_a_ = 0.25 eV for ZGGO:Cr^3+^,Eu^3+^. The probability that a nonradiative transition occurs per unit time (*α*) can be defined according to Eq. (2) as follows [29]:

2 $$ \alpha =s\ \exp \left(-\frac{E_{\mathrm{a}}}{kT}\right) $$

where *s* is the frequency factor (s^−1^), *k* is the Boltzmann constant, and *T* is the temperature. It can be seen that a lower activation energy (*E*_a_) leads to a greater probability (*α*) of a nonradiative transition. Because of the similar activation energy, ZGGO:Cr^3+^ and ZGGO:Cr^3+^,Eu^3+^ exhibited closed thermal stability, indicating Eu^3+^ incorporation did not significantly affect the thermal stability. However, associated phonon side bands (PSBs) centered at 670 nm became dominant at a higher temperature, thus inducing enhanced emission peaks.

**Fig. 9:**
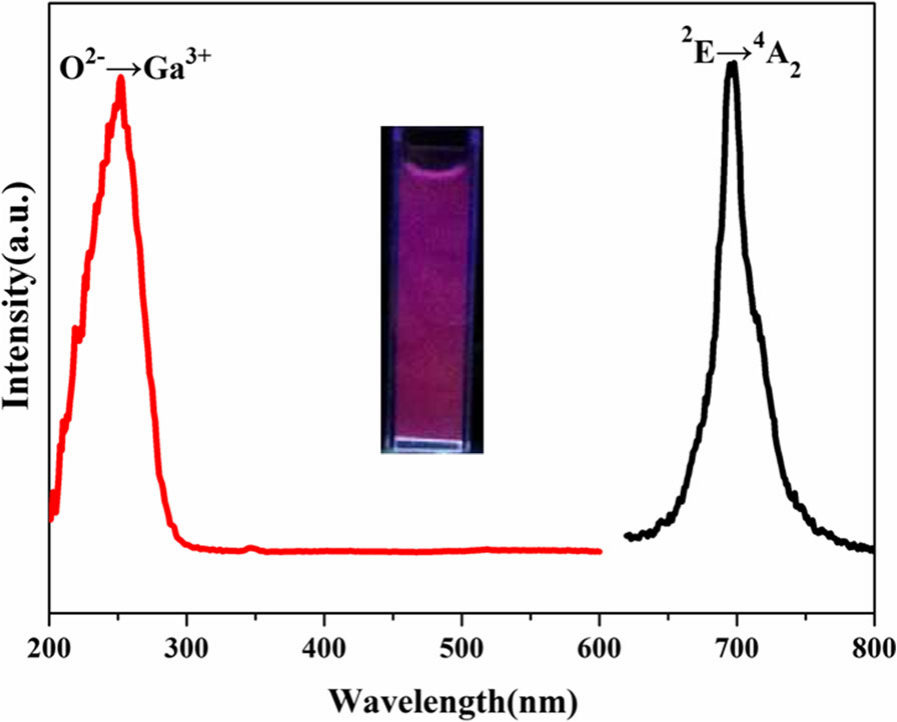
Excitation and emission spectra of ZGGO:Cr^3+^,Eu^3+^ aqueous solution at room temperature. The inset shows the digital photos of ZGGO:Cr^3+^,Eu^3+^ aqueous solution under irradiation of 254 nm UV light

We also investigated the PL excitation and emission spectra of the aqueous dispersion of ZGGO:Cr^3+^,Eu^3+^ (Fig. 9). Compared to the ZGGO:Cr^3+^,Eu^3+^ powder, the aqueous dispersion exhibited almost the same profile of the PL excitation and emission curves except the relatively weak excitation intensity at 300 and 600 nm. The weakened intensity is probably due to the quenching effect of the O–H vibration of water.

**Fig. 10:**
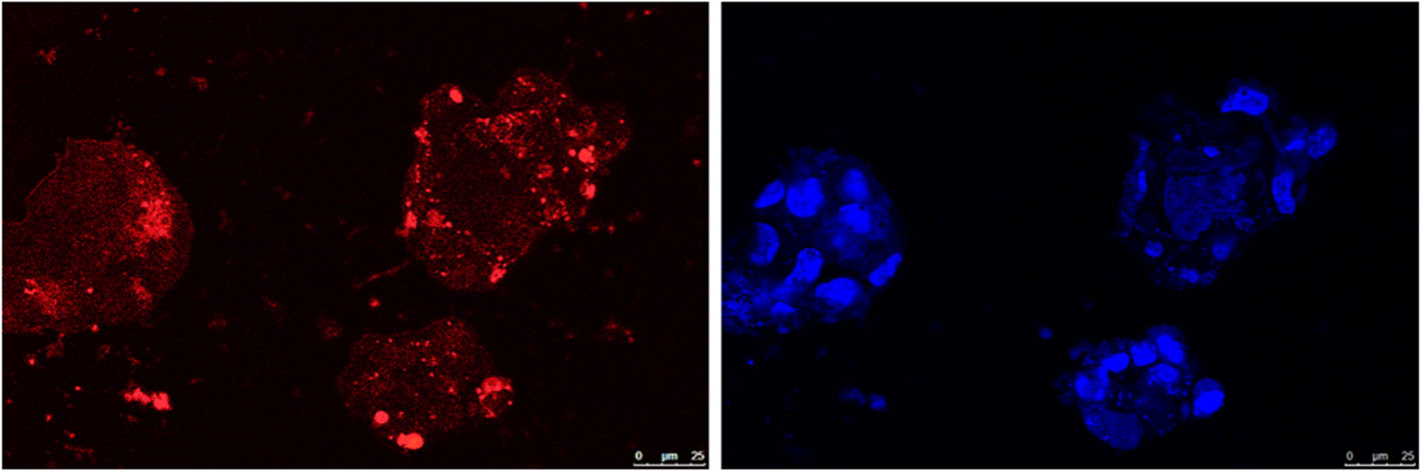
LSCM image (left, red color) of Hek293T cells incubated with ZGGO:Cr^3+^,Eu^3+^-NH_2_ nanoparticles for 1 h. The right image (blue color) is appearance of the same cells stained with 0.5 mL DAPI dye. Scale bar = 25 μm

Hek293T cells were employed here for in vitro imaging test. The as-obtained ZGGO:Cr^3+^,Eu^3+^-NH_2_ nanoparticles were dispersed in cell medium (50 mg/mL), which were excited for 10 min by a 254-nm UV lamp and then moved to culture dishes treated for 1 h. Figure 10 (left, red color) shows the cell luminescence imaging collected on a laser scanning confocal microscope in the absence of excitation. The afterglow luminescence signal of the Hek293T cells was still strong enough to be precisely measured after 1 h, though the afterglow luminescence signals gradually decreased over time. For comparison, the cell luminescence imaging was collected on a laser scanning confocal microscope by another mode from the same cells stained with 0.5 mL DAPI dye (right in Fig. 10, simultaneous excitation). The similar imaging signals suggested that the ZGGO:Cr^3+^,Eu^3+^-NH_2_ nanoparticles possessed an excellent imaging capacity for cells in vitro.

## Conclusions

In this work, spinel structured ZGGO:Cr^3+^ and ZGGO:Cr^3+^,Eu^3+^ nanoparticles were synthesized by a sol-gel method in combination with a subsequent reducing atmosphere-free calcinations. The samples were investigated via detailed characterizations by combined techniques of XRD, TEM, STEM, SAED, PLE/PL spectroscopy, and temperature-dependent PL analysis. The nanoparticles with uniform cubic shape and lateral size of ~ 80–100 nm are of single crystalline and homogeneous solid solution. Excitation of the powder at 273 nm gave a NIR emission band at 697 nm due to the ^2^E → ^4^A_2_ transition in distorted Cr^3+^ ions in gallogermanate, in the absence of Eu^3+^ emission. NIR persistent luminescence of the ZGGO:Cr^3+^,Eu^3+^ can last longer than 7200 s and still hold intense intensity. The persistent luminescence intensity of ZGGO:Cr^3+^ and the afterglow time increase with the incorporation of Eu^3+^ ion. However, Eu^3+^ incorporation did not significantly affect the thermal stability. Finally, the as-obtained ZGGO:Cr^3+^,Eu^3+^-NH_2_ nanoparticles possessed an excellent imaging capacity for cells in vitro.
